# Optical imaging provides flow-cytometry–like single-cell level analysis of HIF-1*α*-mediated metabolic changes in radioresistant head and neck squamous carcinoma cells

**DOI:** 10.1117/1.BIOS.2.1.012702

**Published:** 2025-01-28

**Authors:** Jing Yan, Carlos Frederico Lima Goncalves, Pranto Soumik Saha, Cristina M. Furdui, Caigang Zhu

**Affiliations:** a University of Kentucky, Department of Biomedical Engineering, Lexington, Kentucky, United States; b Wake Forest University, Department of Internal Medicine, Winston-Salem, North Carolina, United States

**Keywords:** optical metabolic imaging, fluorescence microscopy, tumor metabolism, head and neck squamous cell carcinoma, radiation therapy

## Abstract

**Significance:**

Radioresistance remains a significant problem for head and neck squamous cell carcinoma (HNSCC) patients. To mitigate this, the cellular and molecular pathways used by radioresistant HNSCC that drive recurrence must be studied.

**Aim:**

We aim to demonstrate optical imaging strategies to provide flow cytometry–like single-cell level analysis of hypoxia-inducible factor 1-alpha (HIF-1α)-mediated metabolic changes in the radioresistant and radiosensitive HNSCC cells but in a more efficient, cost-effective, and non-destructive manner. Through both optical imaging and flow cytometry studies, we will reveal the role of radiation-induced HIF-1α overexpression and the following metabolic changes in the radioresistance development for HNSCC.

**Approach:**

We optimized the use of two metabolic probes: 2-[N-(7-nitrobenz-2-oxa-1, 3-diazol-4-yl) amino]-2-deoxy-D-glucose (2-NBDG) (to report glucose uptake) and Tetramethylrhodamine ethyl ester (TMRE) (to report mitochondrial membrane potential) with both a standard fluorescence microscope and a flow cytometry device, to report the changes in metabolism between radioresistant (rSCC-61) and radiosensitive (SCC-61) HNSCC cell lines under radiation stresses with or without HIF-1α inhibition.

**Results:**

We found that the matched HNSCC cell lines had different baseline metabolic phenotypes, and their metabolism responded differently to radiation stress along with significantly enhanced HIF-1α expressions in the rSCC-61 cells. HIF-1α inhibition during the radiation treatment modulates the metabolic changes and radio-sensitizes the rSCC-61 cells. Through these studies, we demonstrated that a standard fluorescence microscope along with proper image processing methods can provide flow cytometry–like single-cell level analysis of HIF-1α-mediated metabolic changes in the radioresistant and radiosensitive HNSCC cells.

**Conclusions:**

Our reported optical imaging strategies may enable one to study the role of metabolism reprogramming in cancer therapeutic resistance development at the single-cell level in a more efficient, cost-effective, and non-destructive manner. Our understanding of radiation resistance mechanisms using our imaging methods will offer opportunities to design targeted radiotherapy for improved treatment outcomes for HNSCC patients.

Statement of Discovery We demonstrate that optical imaging strategies can provide flow-cytometry–like single cell level analysis of HIF-1*α* mediated metabolic changes in the radioresistant and radiosensitive HNSCC cells, but in a more efficient, cost-effective, and non-destructive manner. We found that the matched HNSCC cell lines had different baseline metabolic phenotypes, and their metabolism responded differently to radiation stress along with significantly enhanced HIF-1*α* expressions in the radioresistant HNSCC cells. HIF-1*α* inhibition during the radiation treatment modulates the metabolic changes and radiosensitizers the radioresistant HNSCC cells.

## 1 Introduction

Head and neck squamous cell carcinomas (HNSCCs) represent the sixth most common malignancy worldwide,^1^ and the overall 5-year survival rate is ∼50%.^2^ Depending on the stage and resectability, treatment options available for HNSCC patients include various combinations of surgery, radiotherapy (RT), and chemotherapy. RT alone or combined with chemotherapy has been used as a primary curative treatment prescribed for locally advanced HNSCC patients either as definitive or as adjuvant post-surgical therapy.^3^ Statistical data reported that more than 75% of the locally advanced HNSCC patients in the US receive RT as part of their care; however, over 50% of these RT-treated patients are prone to develop recurrence post-treatment,^3^ which leads to most deaths for the HNSCC patients.^4^ Given that HNSCC patients have a high risk for recurrence post-RT, it is crucial to understand the cellular and molecular pathways that support the selective or acquired survival of these cells *in vivo*, with an eventual goal of designing therapies to prevent tumor recurrence and improve the survival of HNSCC patients.

Increasing evidence shows that metabolic reprogramming may be responsible for radioresistance development.^5^ The tumor metabolic rewiring not only provides an unparalleled advantage to tumor cells to survive, grow, and metastasize under a hypoxic and nutrient-poor environment but also endows these cells with plasticity to adapt and escape immunosuppression and therapeutic treatment.^6^^,^^7^ Several groups reported that increased glucose metabolism is associated with radioresistance in breast cancers^8^ and lung cancers.^9^ Some other studies demonstrated that deregulated mitochondrial metabolism is also closely related to the radioresistance development in human cancers.^10^ Recent *in vitro* studies showed that radioresistant HNSCC cells have enhanced glycolysis and decreased oxidative phosphorylation (OXPHOS) compared with radiosensitive HNSCC cells.^11^^–^^13^ Hypoxia-inducible factor-1α (HIF-1α), a master regulator of cellular oxygen sensing and adaptation to hypoxia, plays an essential role in tumor cell survival, growth, and spread.^14^ HIF-1α can be stabilized by hypoxia and also by reactive oxygen species (ROS)^15^^–^^17^ such as those produced from RT.^14^ Previous studies reported that RT induced HIF-1α expression in radioresistant HNSCC cell lines.^18^ HIF-1α overexpression is known to enhance glycolysis and the Warburg effect, promote cancer stem cell-like characteristics, and boost treatment resistance^19^ and has also been shown to increase tumor angiogenesis to facilitate tumor cell proliferation, spread, and treatment resistance.^20^ Taking together, the HIF-1α-mediated changes in metabolism may promote tumor radioresistance and recurrence, targeting the HIF-1α, and the corresponding regulated metabolic changes will offer new opportunities to improve RT efficacy.^21^

In the studies described above, Seahorse assay or metabolomic analysis was used to quantify OXPHOS and glycolysis of tumor cells *in vitro*. Seahorse Assay^22^ and metabolomics^23^ provide valuable measurements of the metabolic phenotypes of tumor cells; however, they are limited to *in vitro* or *ex vivo* studies and cannot be used for repeated time course measurements due to their destructive nature. Although imaging tools such as PET^24^ or MRI^25^ complement the Seahorse Assay and metabolomics for live metabolic scanning, they are not suitable for cellular-level metabolic imaging due to low resolution or low sensitivity.^26^ Flow cytometry has also been extensively used to report cell metabolism using fluorescent probes at the single-cell level^27^ although it is a destructive technique with using expensive equipment. To overcome these limitations, optical imaging has emerged as a new strategy for the non-destructive analysis of multiple metabolic pathways in live cells. Optical measures of two endogenous tissue fluorophores, reduced nicotinamide adenine dinucleotide (NADH) and flavin adenine dinucleotide (FAD)^28^ have been explored to report the reduction-oxidation (redox) state in the electron transport chain of cancer cells.^9^^,^^29^^–^^31^ To quantify tumor glycolysis and mitochondrial function directly and explicitly, we have explored several fluorescent probes for metabolic measurement. The 2-[N-(7-nitrobenz-2-oxa-1,3-diazol-4-yl)amino]-2-deoxy-D-glucose (2-NBDG) has been used in tumors to report glucose uptake,^32^^,^^33^ similar to the clinically available FDG-PET. Tetramethylrhodamine ethyl ester (TMRE) has been utilized to quantify mitochondrial membrane potential (MMP) to study OXPHOS.^34^^,^^35^ Along with novel imaging processing methods, a standard fluorescence microscope may have the potential to provide flow cytometry–like single-cell level analysis of cell metabolism but in a more efficient, cost-effective, and non-destructive manner.

In this study, we demonstrate the use of two probes: 2-NBDG and TMRE with both a standard fluorescence microscope (ZOE™, Bio-Rad) and a flow cytometry device (BD Symphony A3), to report the changes in metabolism between radioresistant (rSCC-61) and radiosensitive (SCC-61) HNSCC cells under radiation stresses with or without HIF-1α inhibition.^36^ Our studies found that rSCC-61 cells have increased baseline glucose uptake and decreased baseline MMP compared with SCC-61 cells. Radiation treatment further enhanced glucose uptake for rSCC-61 cells but not for SCC-61 cells. Moreover, radiation treatment increased MMP for SCC-61 cells but not for rSCC-61 cells. We also observed that radiation induced overexpression of HIF-1α in rSCC-61 cells but not in SCC-61 cells. HIF-1α inhibition in the two cell lines during the radiation treatment modulates their metabolic changes and radio-sensitizes the rSCC-61 cells, which suggested that the radiation induced HIF-1α expression and the following metabolic changes may contribute to the radioresistance development in HNSCC. Through these studies, we also demonstrated that a standard fluorescence microscope along with proper imaging processing software (CellProfiler) can provide flow cytometry–like single-cell level analysis of HIF-1α-mediated metabolic changes in the radioresistant HNSCC cells, but in a more efficient, cost-effective, and non-destructive manner. This study reports the functional flexibility of our optical approach to report the key metabolic changes of radioresistant and radiosensitive HNSCC under therapeutic stress, thereby revealing the role of metabolism reprogramming in the development of resistance to cancer therapeutics. Using this optical metabolic imaging approach, we aim to create a non-destructive platform that can ultimately be translated to *in vivo* imaging using orthotropic HNSCC tumor models^37^ to study the radioresistance development mechanisms at single-cell level resolution.

## 2 Materials and Methods

### 2.1 Cell Culture and Radiation Treatment with HIF-1*α* Inhibition

A matched model of radioresistance for HNSCC including SCC-61 and rSCC-61 cell lines was used in this study.^12^ The SCC-61 cell line that was previously derived from a HNSCC tumor located at the base of the tongue is radiation sensitive, whereas the rSCC-61 cell line generated from SCC-61 cells is radioresistant.^11^ The generation of the SCC-61/ rSCC-61 matched model was described earlier in detail.^11^ Both SCC-61 and rSCC-61 cells used in this study were cultured in the DMEM/F12 medium (Gibco, Waltham, Massachusetts, United States) supplemented with 10% fetal bovine serum (FBS) (Gibco) and 1X penicillin streptomycin (Gibco) at 37°C and 5% ${\mathrm{CO}}_{2}$. rSCC-61 cells were maintained with further weekly radiation of 2 Gy. Cell medium was replaced every 2 days with fresh medium. Where applicable, an X-RAD 225XL orthovoltage preclinical irradiator was used for radiation treatment. In general, ∼75% of confluent cells were exposed to a total of 4 Gy radiation for all radiation treatments. In the HIF-1α inhibition treatment, 50-μM YC-1 in DMSO (Abcam) or equal volume of DMSO as control was added to the medium before the radiation treatment based on former publications.^31^^,^^38^ After radiation and/or HIF-1α inhibition treatments, all cells were then returned to the ${\mathrm{CO}}_{2}$ incubator for 24 h prior to any metabolic imaging, survival test, or western blotting experiments. A single dose of 4 Gy was used in our study as the former dose–response study showed that the SCC-61 and rSCC-61 cells would have significantly different survival rates until a single treatment dose reached up to 4 Gy.^11^ A former time-response study showed that HIF-1α protein expression induced by a 3 Gy radiation treatment will be significantly enhanced and stabilized in radioresistant HNSCC cells at 8 and 24 h post-radiation treatment.^18^ On the other hand, another time-response study showed that the radiation-induced metabolic changes in cells would occur around 24 h post-radiation treatment.^39^ Taken together, we selected the time point of 24 h post-radiation treatment as the time point for our metabolic imaging, survival test, or western blotting experiments.

### 2.2 Survival Test and Western Blotting Experiment

To evaluate the cell survival rate after any treatment, a crystal violet colorimetric test^40^ was performed to test the cell viability. In brief, cells from each experimental group were first fixed with 4% paraformaldehyde for 15 min at room temperature, and then, the fixed cells were washed two times with PBS (1X, Gibco) and exposed to 0.5% crystal violet solution. After 15 min of exposure, excess crystal violet was removed, and cells were washed three times with distilled water. The content of the wells was dissolved in DMSO and read at 565 nm using the Synergy HTX Multi-Mode Microscope Reader (BioTek, Winooski, Vermont, United States). Protein extraction and western blot analysis were performed to characterize HIF-1α expression. The cells were lysed in cold RIPA buffer (Sigma-Aldrich, St. Louis, Missouri, United States) supplemented with a 1X protease inhibitor kit (Roche, Basel, Switzerland) and 1X phosphatase inhibitor (Roche). After extraction, the protein concentration of each sample was determined using the PierceTM BCA Protein Assay kit (Thermofisher Scientific, Waltham, Massachusetts, United States). All protein samples were heated in boiling water after adding 1X Laemmli Sample Buffer (Bio-Rad, Hercules, California, United States) and 5% 2-Mercaptoethanol (Bio-Rad). 30-μg protein was resolved on an 8% to 15% (depending on the molecular weight of the protein of interest) SDS-polyacrylamide gel (PAGE) (Bio-Rad) and transferred to nitrocellulose (NC) membrane (Bio-Rad). Membranes were incubated in a blocking solution (5% non-fat dried milk dissolved in Tris-buffered saline). HIF-1α (1:1000 dilution, 36169, Cell Signaling Technology, Danvers, Massachusetts, United States) and beta-actin (1:2000 dilution, MA1-140, Invitrogen, Waltham, Massachusetts, United States) were diluted in blocking solutions. The membranes were washed and incubated with appropriate secondary antibodies and assessed by SuperSignal™ West Pico PLUS Chemiluminescent Substrate (Thermofisher Scientific™) on Tanon 5200 Chemiluminescent Imaging System. All bands were normalized to the 45-kDa beta-actin band as a loading control.

### 2.3 Optical Metabolic Imaging and Flow Cytometry Experiments

Optical metabolic imaging and flow cytometry of glucose uptake and MMP on the HNSCC cells were conducted using our previously validated fluorescence probes including 2-NBDG and TMRE. The fluorescent probes including 2-NBDG and TMRE were chosen for our imaging due to their translatability to *in vivo* experiments as published by us before.^33^^,^^35^ The 2-NBDG (Biogems, Westlake Village, California, United States) is an optical analog glucose that enters the cell via glucose transporters,^41^^–^^43^ which measures glucose uptake analogous to widely accepted PET imaging.^44^^,^^45^ TMRE (Biotium, Fremont, California, United States) is a cationic dye that accumulates in the mitochondrial inner membrane as a function of MMP,^46^ which has been utilized extensively to study mitochondrial metabolic capability.^33^^,^^35^ During labeling, 2-NBDG and TMRE were diluted to final concentrations of 200  μmol/L and 50  nmol/L, respectively, in glucose-free DMEM (Gibco) with 10% dialyzed FBS (Gibco) and 1X Penicillin Streptomycin (Gibco). Concentrations were chosen based on standard manufacturer protocols to not affect the metabolism of cells and minimize each probe’s toxicity.^42^ Before any optical imaging or flow cytometry, the regular media was removed, and the cell was washed with PBS and incubated at 37, 5% ${\mathrm{CO}}_{2}$ for 30 min with either 200  μmol/L 2-NBDG or 50  nmol/L TMRE dissolved in glucose-free cell media. Cells were only stained with one of two probes to prevent metabolite competition and minimize optical and biological cross-talk between metabolic probes.^42^ All optical metabolic images were collected using a fluorescence microscope (ZOE™ Fluorescent Cell Imager, Bio-Rad). The green channel that has an excitation peak of 488 nm (17-nm bandwidth) and emission peak of 517 nm (23-nm bandwidth) was used for 2-NBDG uptake imaging, and the red channel with an excitation peak of 556 nm (20-nm bandwidth) and emission peak of 615 nm (61-nm bandwidth) was used for TMRE uptake imaging. The gain level, contrast level, and LED intensity of the microscope were optimized and then fixed for either 2-NBDG or TMRE imaging at all different time points to ensure the images with sufficient optical signals could be obtained and compared. The optical metabolic microscope has a 20× objective, a field of view of $0.70\;mm^{2}$, with a resolution of ∼1  μm (∼2.7  pixels). The cells were cultured in a 12-well plate, and the cell density was ∼0.2 to $0.5\times 10^{6}\;\text{cells}$ per well. These factors can significantly influence the performance of cell segmentation algorithms. Higher-quality imaging of cells with appropriate cell density can improve segmentation accuracy; improper conditions may lead to difficulties in distinguishing individual cells. In flow cytometry experiments, the cells were detached by 0.25% Trypsin (Gibco) after probe labeling and then pelleted at 1500 rpm for 5 min by a centrifuge (Fisher Scientific, Hampton, New Hampshire, United States). To maximize the cell viability and signal intensity, the cell pellets were directly resuspended with cold PBS and then analyzed using a flow cytometer (BD Symphony A3, BD Bioscience, Franklin Lakes, New Jersey, United States) with optimized configurations. Specifically, 2-NBDG was excited by a blue laser (488 nm) and detected with a filter set of 505-nm long pass dichroic filter and 515  nm±20  nm band pass emission filter, whereas TMRE was excited by a yellow-green laser (561 nm) and detected with a filter set of 570-nm long pass dichroic filter and 586  nm±15  nm bandpass emission filter. DAPI was excited by an ultraviolet laser (355 nm) and detected with a filter set of 410-nm long pass dichroic filter and 450  nm±50  nm bandpass emission filter to exclude dead cells. Flow cytometry data were collected and processed using BD FACSDIVATM and FlowJo software provided by the Flow Cytometry and Immune Monitoring Core Facility at the University of Kentucky.

### 2.4 Optical Metabolic Imaging Data Analysis

**Fig. 1:**
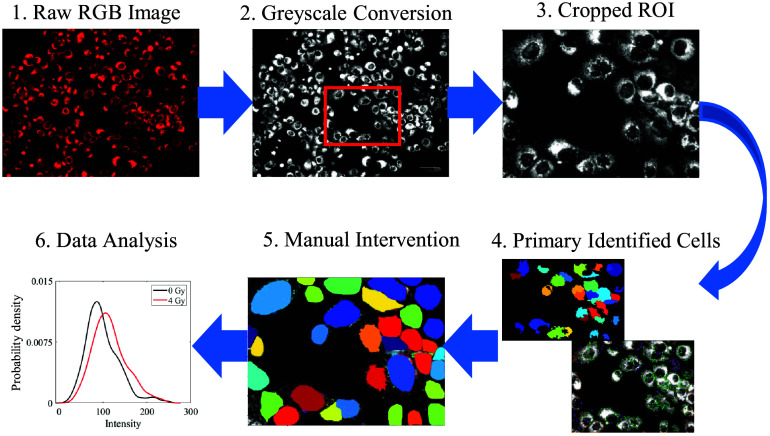
Optical microscope along with proper image processing software can characterize metabolism per cell.

To provide flow cytometry–like single-cell data analysis for optical metabolic imaging, all 2-NBDG and TMRE images at different experimental groups were processed, as illustrated in Fig. 1. To be brief, we utilized publicly available open-source software CellProfiler (version 4.2.6) to analyze fluorescent microscopy images measured on *in vitro* HNSCC cells. To identify individual cells from fluorescence images, we initially utilized the automated ‘primary object identification’ toolbox of the cell profiler, followed by manual object identification to improve cell identification accuracy. In the primary object identification toolbox, the diameter range of the identified cells was chosen to be between 50 and 100-pixel units (18 to 38  μm) using global minimum cross-entropy thresholding as it provided the highest accuracy. Cells outside of this range were manually identified using the manual object identification toolbox in CellProfiler. The intensity distribution histograms were generated by calculating the mean intensity values of individual identified cells from each image, considering the whole cell region without isolating the nucleus. The mean intensity values of each identified cell from all the images were then saved in an Excel sheet. By default, CellProfiler outputs the individual cell intensity values within the range of 0 to 1 for the identified cells (single-cell level analysis), we have then converted each cell intensity distribution value to 8-bit by multiplying the intensity values with 255. To speed up the processes, random 1000×750-pixel windows were cropped from each image for individual cell intensity analysis. For a comparison among the different experimental groups, 10 to 20 random images from each experimental group were selected and processed using the aforementioned method. Once all images were processed by the Cell Profiler, MATLAB (MathWorks, R2023a, Natick, Massachusetts, United States) software was used to provide flow cytometry–like data analysis. Specifically, the kernel smoothing function (ksdensity) and kernel distribution fit were used to generate the frequency plot and then to provide the histogram analysis for the fluorescence intensity. The mean intensities for each experimental group were also created and compared using Student’s t-test (between two groups) or analysis of variance (ANOVA) test (among three groups). p values<0.05 were considered statistically significant. MATLAB Statistics Toolbox was used for all statistical tests.

To quantitatively compare the optical imaging data with flow cytometry results, the histograms generated from optical images and flow cytometry data were evaluated and compared with several metrics. Specifically, the relative changes of histogram peak locations, histogram FWHM (full width at half maximum), mean intensities, and median intensities among different experimental groups were calculated and compared. The p-values were also summarized and compared between the optical imaging data and flow cytometry results.

## 3 Results

### 3.1 Optical Metabolic Imaging and Flow Cytometry Capture Increased Glucose Uptake and Decreased MMP in rSCC-61 Cells Versus SCC-61 Cells

**Fig. 2:**
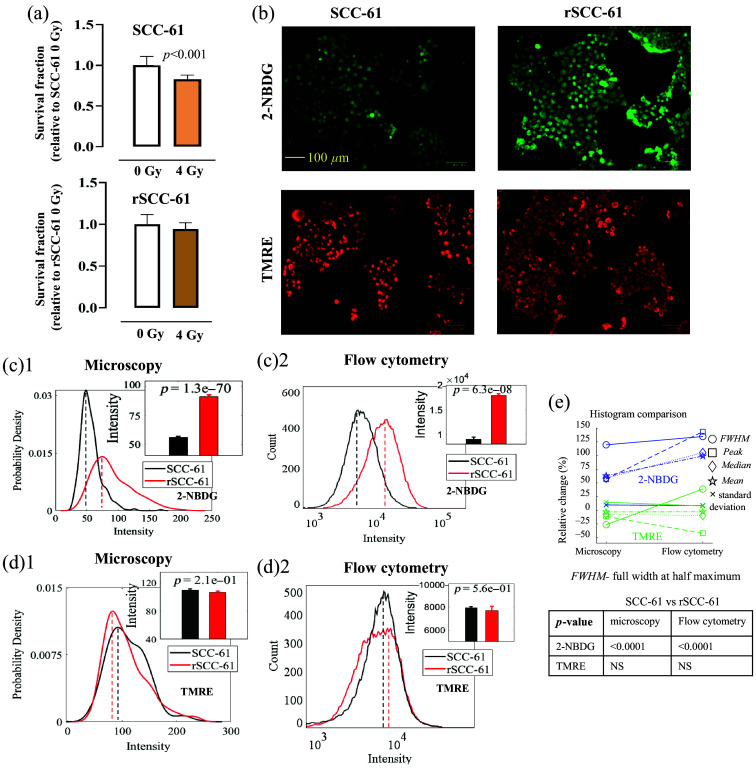
rSCC-61 cells had increased glucose uptake and decreased MMP compared with SCC-61 cells. (a) Survival fractions of rSCC-61 and SCC-61 cells under 4 Gy of radiation treatment. (b) Representative fluorescent imaging shows that the rSCC-61 cells had increased 2-NBDG uptake and decreased TMRE uptake compared with SCC-61 cells. Statistical analysis of HNSCC baseline glucose uptake (c)1 and MMP (d)1 based on metabolic images. Statistical analysis of HNSCC baseline glucose uptake (c)2 and MMP (d)2 based on flow cytometry data. (e) Histogram characteristic changes between the histograms generated from optical images and flow cytometry data. Peak refers to the x-axis value (intensity) indicated by the dashed lines (probability density peak or count peak) in panels (c)1, (c)2, (d)1, and (d)2. Median refers to the median intensity of all cell populations, whereas mean refers to the mean intensity of all cell populations. The sample size for optical imaging was 10 to 20 (images) per group. The sample size for flow cytometry was three to six samples per group. The sample size for the survival test was 12 per group. Student’s t-test was used for statistical analysis.

To compare the baseline metabolic phenotypes of rSCC-61 cells and SCC-61 cells, both optical metabolic imaging and flow cytometry were conducted on these two HNSCC cell lines. Figure 2 shows the glucose uptake (2-NBDG uptake) and MMP (TMRE uptake) of radioresistant (rSCC-61) and radiosensitive (SCC-61) HNSCC cells characterized by both optical metabolic imaging and flow cytometry. Figure 2(a) shows the survival rates of the two HNSCC cell lines under 4 Gy of radiation treatment. The survival rate data confirmed that rSCC-61 cells are more radioresistant compared with SCC-61 cells. The representative fluorescence imaging shows that rSCC-61 cells had increased glucose uptake but decreased MMP compared with SCC-61 cells [Fig. 2(b)]. Figure 2(c)1 shows the statistical analysis using the optical imaging data collected on different batches of experiments and illustrates that the glucose uptake level in rSCC-61 cells was statistically higher compared with SCC-61 cells (p<0.0001). By contrast, Fig. 2(d)1 shows that the MMP of rSCC-61 cells was lower than that in SCC-61 cells but not statistically significant. Figures 2(c)2 and 2(d)2 show the corresponding flow cytometry data measured on the same batch of HNSCC cells. The flow cytometry results also show that rSCC-61 cells had significantly higher glucose uptake [Fig. 2(c)2] but slightly lower MMP [Fig. 2(d)2] compared with SCC-61 cells. Both optical imaging and flow cytometry results are consistent with the previous analysis of energy metabolism in these two HNSCC cell lines using Seahorse Assay.^12^ Figure 2(e) shows the histogram characteristics changes for the histograms generated from optical images and flow cytometry data. Generally, the histogram characteristics changes between the optical data and flow cytometry data showed that the metabolic changes between the two HNSCC cell lines are consistent although it appears that the histograms from flow cytometry had larger changes among different experimental groups compared with that from optical imaging, which suggested that flow cytometry has a higher sensitivity compared with optical microscopy in our study.

### 3.2 rSCC-61 Cells Had Different Metabolic Changes Under Radiation Stress Compared with SCC-61 Cells, Along with Enhanced HIF-1*α* Expression in rSCC-61 Cells

**Fig. 3:**
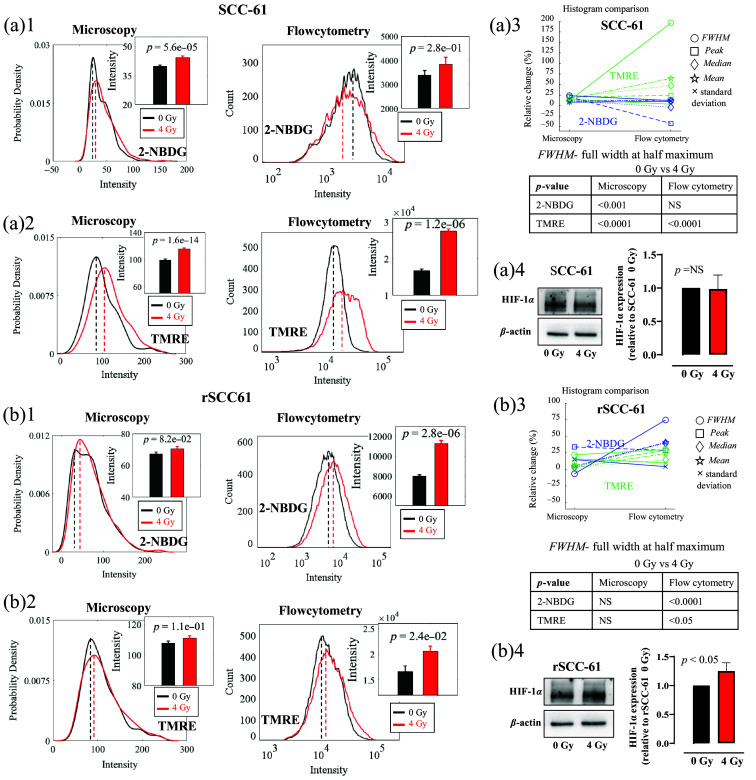
rSCC-61 cells had different metabolic changes under radiation stress compared with SCC-61 cells, along with enhanced HIF-1α expression in rSCC-61 cells but not in SCC-61 cells. The 2-NBDG uptake and TMRE uptake changes were acquired from both optical metabolic images and the corresponding flow cytometry experiments. Statistical analysis of the 2-NBDG uptake changes post radiation treatment for SCC-61 cells (a)1 and rSCC-61 cells (b)1. Statistical analysis of the TMRE uptake changes post radiation treatment for SCC-61 cells (a)2 and rSCC-61 cells (b)2. (a)3 and (b)3 Histogram characteristic change between the histograms generated from optical images and flow cytometry data. Peak refers to the x-axis value (intensity) indicated by the dashed lines (probability density peak or count peak) in panels (a)1, (a)2, (b)1, and (b)2. Median refers to the median intensity of all cell populations, whereas mean refers to the mean intensity of all cell populations. Representative western blotting images and the statistical analysis of HIF-1α expression in SCC-61 cells (a)4 and rSCC-61 cells (b)4 under 4 Gy radiation treatment. The sample size for imaging was 10 to 20 (images) per group. The sample size for flow cytometry was 3 to 4 samples per group. The sample size for western blotting was four repeats (six data points in total) per group. Student’s t-test was used for statistical analysis.

To investigate the metabolic responses under radiation stresses, both SCC61 and rSCC-61 cells were irradiated with a 4 Gy radiation and then characterized by both optical metabolic imaging and flow cytometry. Figure 3 shows glucose uptake and MMP of SCC-61 cells and rSCC-61 cells with or without radiation treatment. The glucose uptake and MMP were quantified based on both optical fluorescence images and flow cytometry data. The histograms in Figs. 3(a)1 and 3(b)1 illustrate that radiation stress enhanced glucose uptake in rSCC-61 cells but not in SCC-61 cells. However, the average intensities in the bar figures showed that the radiation treatment upregulated the glucose uptake in both rSCC-61 and SCC-61 cells. Similarly, the histograms in Figs. 3(a)2 and 3(b)2 illustrate that the MMP was increased post-radiation treatment for SCC-61 cells but not for rSCC-61 cells, whereas the average TMRE uptake intensities showed that the radiation enhanced MMP for both cell lines. Figures 3(a)3 and 3(b)3 show the histogram characteristics changes for the histograms generated from optical images and flow cytometry data. Overall, the histogram characteristics changes between the optical data and flow cytometry data showed that the metabolic changes between the control group (0 Gy) and RT treatment group (4 Gy) are consistent for both SCC-61 cells [Fig. 3(a)3] and rSCC-61 cells [Fig. 3(b)3], whereas the histograms from flow cytometry had larger changes among different experimental groups compared with that from optical imaging, further suggesting that flow cytometry has a higher sensitivity compared with optical microscopy in our study. Figure 3 shows that the statistical analysis of the cell metabolism changes using metabolic images is always consistent with that using the flow cytometry data. To explore if the HIF-1α protein was involved in the metabolic changes under the radiation stress, western blotting experiments were also conducted on the same batch of HNSCC cell lines with or without radiation treatment. The western blotting results in Figs. 3(a)4 and 3(b)4 show that the HIF-1α expression was significantly enhanced in rSCC-61 cells (p<0.05) under radiation stress but not in SCC-61 cells, which suggests that HIF-1α may be associated with metabolic changes in the acquisition of radioresistance in HNSCC cells.

**Fig. 4:**
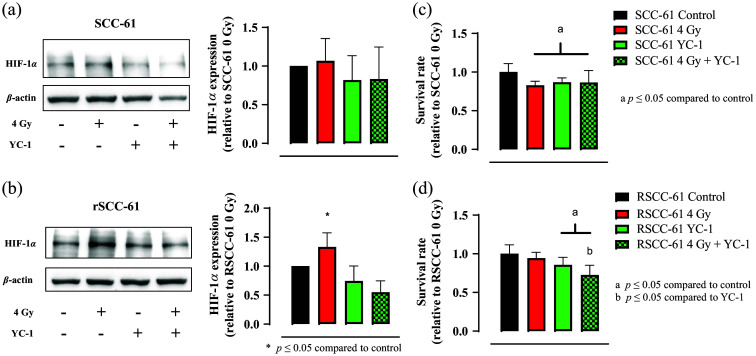
Radiation induced overexpression of HIF-1α in rSCC-61 cells but not in SCC-61 cells, and the HIF-1α inhibition radio-sensitizes the rSCC-61 cells. Representative western blotting images and the statistical analysis of HIF-1α expression in SCC-61 cells (a) and rSCC-61 cells (b) under different treatments. The radiation treatment induced and enhanced HIF-1α in both rSCC-61 and SCC-61 cells but was only statistically significant for rSCC-61 cells. HIF-1α inhibition mitigates the HIF-1α inhibition expressions in both rSCC-61 and SCC-61 cells but was only statistically significant for rSCC-61 cells. The corresponding survival rates of SCC-61 cells (c) and rSCC-61 cells (d) under different treatments. All results were normalized to their corresponding control group. The sample size for all western blotting experiments was five repeats (seven data points in total) per group. The sample size for the survival test was 12 per group. Student’s t-test was used for statistical analysis.

### 3.3 Radiation Induces Significantly Increased HIF-1*α* Expression in rSCC-61 Cells and the HIF-1*α* Inhibition Radio-Sensitizes the rSCC-61 Cells

To further reveal the role of radiation-induced HIF-1α expression in the HNSCC radioresistance development, we further conducted western blotting experiments on rSCC-61 and SCC-61 cells with various treatments, including no treatment (control), 4 Gy radiation-only treatment, HIF-1α inhibition only, and 4 Gy radiation treatment plus HIF-1α inhibition. Figures 4(a) and 4(b) show the representative western blotting results of HIF-1α expressions in SCC-61 and rSCC-61 cells under the four different treatments, whereas Figs. 4(c) and 4(d) show their corresponding survival rates. Figure 4(a) shows that radiation treatment slightly upregulates the HIF-1α expression in SCC-61 cells, whereas the HIF-1α inhibition mitigates the HIF-1α expression. Figure 4(c) shows that the survival rates of SCC-61 did not change much when the HIF-1α inhibition was introduced in SCC-61 cells compared with those that only received radiation treatment. Figure 4(b) shows that radiation stress significantly upregulates the HIF-1α expression in rSCC-61 cells (p<0.005), whereas the HIF-1α inhibition significantly mitigates the HIF-1α expression (p<0.005). Figure 4(d) shows that the survival rates of rSCC-61 decrease significantly after the HIF-1α inhibition in the radiation treated rSCC-61 cells compared with the rSCC-61 cells that only received radiation treatment (p<0.005). Together, Fig. 4 suggests that HIF-1α may be a potential target to sensitize radiation response in HNSCC.

### 3.4 HIF-1*α* Inhibition Modulates the Metabolic Changes Induced by Radiation Stress in Both rSCC-61 Cells and SCC-61 Cells

**Fig. 5:**
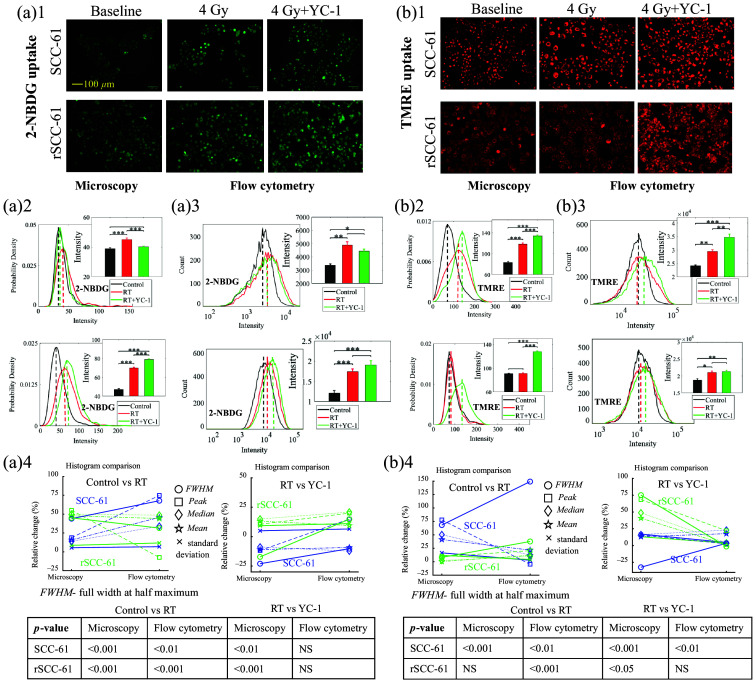
HIF-1α inhibition using YC-1 modulates the metabolic changes induced by radiation stress for rSCC-61 and SCC-61 cells. Representative 2-NBDG uptake imaging (a)1 and TMRE uptake imaging (b)1 at baseline, 4 Gy radiation treatment, and the combination of 4 Gy radiation treatment with HIF-1α inhibition for SCC-61 and rSCC-61 cells. Statistical analysis of 2-NBDG uptake changes among the control group, radiation treatment group, and radiation plus HIF-1α inhibition group using optical imaging data (a)2 and flow cytometry data (a)3. Statistical analysis of TMRE uptake changes among the control group, radiation treatment group, and radiation plus HIF-1α inhibition group using optical imaging data (b)2 and flow cytometry data (b)3. (a)4 and (b)4 Histogram characteristics changes between the histograms generated from optical images and flow cytometry experiments from optical images and flow cytometry experiments. Peak refers to the x-axis value (intensity) indicated by the dashed lines (probability density peak or count peak) in panels (a)2, (a)3, (b)2, and (b)3. Median refers to the median intensity of all cell populations, whereas mean refers to the mean intensity of all cell populations. The sample size for optical imaging was 10 to 20 images per group. The sample size for flow cytometry was three to six samples per group. * represents p<0.05, ** represents p<0.01, and *** represents p<0.001. The ANOVA test was used for statistical analysis. Both the metabolic imaging and flow cytometry experiments were repeated independently at least two times, which all yielded consistent trends for the metabolic changes under various treatments.

To further investigate if the radiation-induced HIF-1α overexpression in the radioresistant rSCC-61 cells was associated with metabolic changes post-radiation treatment, HIF-1α inhibition (YC-1) experiments during the radiation treatment on the two HNSCC cell lines were conducted. Figure 5 shows the metabolic changes of the two cell lines receiving different treatments including control and radiation with or without HIF-1α inhibition. Figure 5(a)1 presents typical 2-NBDG uptake images that reflect changes in the glucose uptake among three different experimental groups, whereas Fig. 5(b)1 shows the typical TMRE uptake images that reflect the MMP changes among the three experimental groups. Figures 5(a)2 and 5(a)3 report the corresponding statistical analysis on the 2-NBDG uptake among groups based on the optical metabolic images and flow cytometry data. Both optical imaging data and flow cytometry results show consistent trends for glucose uptake changes in the two cell lines under various treatments. Specifically, the radiation treatment enhanced glucose uptake in both SCC-61 and rSCC-61 cells compared with their control group, whereas the HIF-1α inhibition reversed the radiation-induced changes in SCC-61 cells but not in rSCC-61 cells. Rather, the HIF-1α inhibition further enhanced glucose uptake in rSCC-61 cells compared with the radiation-treated group. Figures 5(b)2 and 5(b)3 show the statistical analysis on the MMP among different groups based on both optical imaging data and flow cytometry data. Optical imaging data show that radiation treatment enhanced MMP in SCC-61 cells compared with their control group, whereas the HIF-1α inhibition along with radiation treatment further enhanced the MMP in SCC-61 cells compared with the radiation treatment group, as shown in Fig. 5(b)2. By contrast, the optical imaging data shows that radiation treatment did not upregulate MMP in rSCC-61 cells compared with their control group, whereas the HIF-1α inhibition plus radiation stress significantly enhanced the MMP in rSCC-61 cells compared with the radiation treatment group [Fig. 5(b)2]. The flow cytometry results of Fig. 5(b)3 show that the TMRE uptake changes in the two cell lines from different experimental groups are generally consistent with the optical imaging data. Figures 5(a)4 and 5(b)4 show the histogram characteristics changes for the histograms generated from optical images and flow cytometry data. Overall, the histogram characteristics changes between the optical data and flow cytometry data showed that the metabolic changes among the control group (0 Gy), RT treatment group (4 Gy), and RT treatment plus HIF-1α inhibition group (4 Gy+ YC-1) are generally consistent for both HNSCC cell lines.

## 4 Discussion

Interest in tumor metabolism continues to grow in the field of cancer research as metabolic reprogramming of tumors has been recognized as one of the major cancer hallmarks.^47^ Despite a variety of metabolic tools that report on different endpoints to piece together a narrative on metabolic reprogramming of tumor cells that escape therapies, there still exists a significant unmet need for a translational technique with the ability to directly link *in vitro* and *in vivo* results for cancer research. For example, the most commonly used metabolic tool for *in vitro* cell studies is the Seahorse Assay, which perturbs cell metabolism with special chemicals, following which the oxygen consumption and extracellular acidification rates are measured.^48^^–^^57^ Metabolomics can simultaneously screen a large number of metabolites and map metabolic networks from *in vitro* cells and *ex vivo* tissues to even human subjects but requires sample extraction.^23^^,^^58^ Because of their destructive nature, it is impractical to conduct multiple metabolic measurements on the same population of cancer cells or *in vivo* using either Seahorse Assay or metabolomics. FDG-PET^24^ is commonly used for *in vivo* measurement of glucose uptake (FDG-PET), and magnetic resonance spectral imaging^25^^,^^26^ has been explored to report mitochondrial metabolism and glycolysis by monitoring P31 or C13 nuclei in metabolites *in vivo*, but they have either low resolution or low sensitivity. Optical metabolic imaging could potentially fill this critical gap due to its capability to directly measure the metabolic parameters from *in vitro* cells, *ex vivo* tissues, and *in vivo* tumor models at subcellular level resolution in a non-destructive manner.

Autofluorescence of reduced nicotinamide adenine dinucleotide (NADH) and flavin adenine dinucleotide (FAD) have been explored as a non-destructive approach to report the reduction-oxidation (redox) state of cells^29^^,^^30^^,^^59^^,^^60^ by looking at the ratio of the two (FAD/NADH) and then provide an indirect measure of the balance between glycolysis and OXPHOS. FAD- and NADH-based label-free autofluorescence techniques have been explored extensively by others for cancer applications.^29^^,^^30^^,^^59^^,^^60^ However, autofluorescence imaging typically requires expensive two-photon microscopy to avoid photon damage on cells,^61^ and autofluorescence signal from cells is relatively weak. To address some of the limitations associated with the autofluorescence technique, we have exploited metabolic probes-based approaches to measure cell glucose uptake and mitochondrial function directly and explicitly. Specifically, we utilized fluorescence probes such as 2-NBDG and TMRE to measure glucose uptake and mitochondrial function of cells with the use of commonly available single-photon microscopes at a lower cost. These labeling-based techniques are relatively easy to implement and are compatible with live-cell imaging, providing distinct functional information. However, they involve the use of external probes that require a labeling procedure, and their signals are not endogenous, potentially limiting their ability to reflect the natural metabolic state.

To demonstrate the capability of optical techniques, here, we utilized our optical imaging methods along with the flow cytometry technique to capture the metabolic changes of radioresistant and radiosensitive HNSCC cells *in vitro*. For the first time, we demonstrated that a standard fluorescence microscope along with proper imaging processing software (CellProfiler) can provide flow cytometry–like single-cell level analysis of HIF-1α-mediated metabolic changes in the radioresistant HNSCC but in a more efficient, cost-effective, and non-destructive manner. It should be noted that the optical imaging method is theoretically non-destructive under conditions where photon damage is negligible, minimizing any potential perturbations to cell metabolism that could arise from destructive techniques. Moreover, due to its non-destructive nature, the optical technology is suitable for repeatable and longitudinal studies on the same population of *in vitro* cells or patient-derived organoids.^62^ To demonstrate the proof-of-concept, we utilized CellProfiler for single-cell analysis from fluorescence images in this study. CellProfiler has been widely used in the research community as open-source software with a user-friendly interface and a minimal requirement for programming knowledge. CellProfiler offers flexibility to choose from a variety of cell segmentation algorithms, such as threshold algorithms, morphology algorithms, and watershed algorithms, or a combination of these, depending on user needs. It also allows for the customization of parameters to improve segmentation accuracy. However, it should be noted that other open-source software tools, such as Ilastik and Icy, and deep learning-based platforms such as CellPose, DeepCell, and StarDist, can also be employed for single-cell analysis. The performance of these tools may vary depending on the specific dataset, and each has its advantages for different types of analysis.

The classical notion of ineffective radiotherapy is primarily attributed to hypoxia.^63^^,^^64^ However, increasing evidence shows that metabolic reprogramming may also be responsible for the development of radioresistance in cancers.^5^ HIF-1α plays an essential role in tumor cell survival, growth, and metastasis.^14^ On the one hand, HIF-1α regulates the expression of genes involved in OXPHOS and energy production and promotes the Warburg effect,^14^ enhanced glycolysis, and lactate secretion in the presence of oxygen in many proliferating tumor cells. On the other hand, HIF-1α also enhances angiogenesis to facilitate tumor growth.^20^ HIF-1α can be stabilized by hypoxia and also by ROS^15^^–^^17^ produced from RT.^14^ Previous studies reported that the RT induced significantly increased HIF-1α in many types of human cancer cells, including breast cancer,^65^ lung cancer,^9^ and HNSCC.^18^ Former *in vitro* studies of the matched model of radioresistance for HNSCC used in this study (rSCC-61 and SCC-61) showed that radioresistant HNSCC cells have enhanced glycolysis and decreased OXPHOS compared with their parental radiosensitive cells.^11^^,^^12^ Based on the above findings, it is reasonable to hypothesize that the radiation induced HIF-1α overexpression, and the following metabolic changes might be responsible for the development of radioresistance in HNSCC.

In this study, we utilized optical metabolic imaging and a matched model of radiation resistance for HNSCC (SCC-61 and rSCC-61) to understand the role of radiation-induced HIF-1α and the following metabolic changes between the radiosensitive and radioresistant HNSCC cells. Our *in vitro* cell studies found that the radioresistant HNSCC cells have increased baseline glucose uptake and decreased MMP compared with the radiosensitive cells. The optical imaging data are consistent with the flow cytometry data and the published Seahorse Assay results.^11^^,^^12^ We also found that radiosensitive HNSCC cells (SCC-61) have increased glucose uptake and increased MMP under radiation stress. It is widely accepted that mitochondria produce more ROS at high MMP;^66^ thus, the increased MMP in SCC-61 cells suggested a significantly increased ROS induced by RT as reported previously,^11^ which can lead to increased oxidative stress. By contrast, radiation treatment of the radioresistant HNSCC cells (rSCC-61) further enhanced glucose uptake while unchanged MMP along with significantly increased HIF-1α expressions. The unchanged MMP in rSCC-61 cells suggested that radiation treatment did not induce excess ROS production in the rSCC-61 cells, in agreement with the published data.^11^ RT-enhanced glucose uptake in rSCC-61 cells can compensate for the loss of energy production due to unchanged MMP to sustain cell growth under RT. Earlier studies reported that radiation induces HIF-1α overexpression to promote glycolysis in radioresistant lung cancer cells that may help them escape from the RT.^9^^,^^31^ These RT-induced metabolic events in rSCC-61 cells may well be caused by the observed HIF-1α overexpression as HIF-1α inhibition modulated the metabolic changes induced by radiation. Together, these data suggest that the radiation-induced HIF-1α overexpression and associated metabolic changes may contribute to the radiation resistance in rSCC-61 cells. The HIF-1α inhibition significantly promoted the MMP in rSCC-61 cells along with the survival tests shown in Fig. 4(d), which confirmed that the HIF-1α inhibitor (YC-1) could potentially radio-sensitize the HNSCC cells, a finding that supports future therapeutic investigations.^31^ It was interesting to observe that the HIF-1α inhibition further enhanced glucose uptake in rSCC-61 cells in our study. Though we are not fully clear on the mechanism behind this phenomenon yet, these observations may be supported by compensatory or adaptive metabolic mechanisms that reported in other human cancer cells.^67^^,^^68^ For example, a former study reported that inhibiting HIF-1α might activate alternative glycolytic drivers such as c-Myc or AMPK, which can enhance the expression of glucose transporters or enzymes to promote glucose uptake even in the absence of HIF-1α activity.^67^ Another study showed that radioresistant cells can rely on HIF-1-independent adaptations for survival, such as activating pathways involving PI3K-Akt, mTOR, or others, which would compensate for the loss of HIF-1α activity and promote glucose metabolism.^68^ Nevertheless, we will continue to explore the potential mechanism behind this interesting phenomenon in our future study.

The work described here reported an effective imaging strategy for the direct study of two key metabolic endpoints (glucose uptake and MMP) related to tumor metabolism and has the potential to be expanded to image other endpoints such as fatty acid uptake^69^ in our future work. This study demonstrated that a standard fluorescence microscope along with proper image processing software can provide flow cytometry–like analysis of cell metabolism at the single-cell level. It should be noted that the inherent differences between these two techniques pose some challenges for direct quantitative comparisons. However, we still provided some comparisons of the results obtained by these two technologies using multiple matrices, including standard deviation, p-value, histogram peak location changes, mean and median intensity changes, and histogram FWHM changes between experimental groups. These quantitative matrices reveal some variations in the readouts between the two techniques. Specifically, we noticed that the standard deviations for metabolic parameters from fluorescence images were slightly larger than those from flow cytometry, and the relative changes of histogram peak locations, mean and median intensities, and FWHM from fluorescence images were smaller than those from flow cytometry data. However, the p-values among various experimental groups generated from fluorescence images are generally comparable with those from flow cytometry data. These discrepancies in the readouts can be caused by several factors: (1) In flow cytometry experiments, cells were suspended for individual cell intensity readout, whereas microscopy images were taken while retaining the cells in their native conditions; (2) Flow cytometry results were generated using significantly larger cell populations (∼20,000) compared with microscopy imaging (∼1000); and (3) The microscope results were highly dependent on the image quality, microscope configuration, and the choice of cell segmentation software; (4) The flow cytometry is more sensitive compared with the standard fluorescence microscope used in our study. Nevertheless, our results showed that microscopy provided similar trends between experimental groups compared with flow cytometry, but in a more efficient, cost-effective, and non-destructive manner.

The selection of proper techniques for measuring individual cellular metabolism depends on the specific needs of the experiment, as each method has its own advantages and limitations. Flow cytometry measures individual cell intensity in suspension, passing cells sequentially through a laser beam, making it ideal for high-throughput population-based analysis. Optical microscopies capture cellular fluorescence intensities within their native microenvironment, offering the unique advantage of spatial and contextual information that flow cytometry cannot provide. A detailed comparison of these two techniques for cellular metabolism analysis is shown in Table 1 to better understand the trade-offs and make informed decisions.

**Table 1:** Comparison of flow cytometry and microscopy for cellular metabolism analysis.

Metrics	Flow cytometry	Microscopy
Sample types	Cells	Cells, *ex vivo* tissue
Special preparation	Detachment, suspension	None
Sensitivity	Very high	High (nM for TMRE, μM for 2-NBDG)
Signal-to-background ratio	∼10 for 2-NBDG, ∼1000 for TMRE	∼20 for 2-NBDG, ∼80 for TMRE
Throughput	Million cells per test	Thousands of cells per test
Quantification	Statistical	Parametric, can be statistical
Morphological	No	Yes
Complexity	Moderate	Low
Cost in instrumentation	Half million USD	Less 10k USD

It should be noted that tumor cell metabolism is highly dependent on its micro-environment. Although *in vitro* cultures remain critically important in cancer research, they cannot fully replicate the tumor microenvironment, which limits their applications to translation studies. The next step to understand the role of metabolic reprogramming in radioresistance development for HNSCC should be to image *in vivo* the landscape of 2-NBDG and TMRE changes in tumor tissues with relevant vasculature and stromal environment using an orthotropic HNSCC tumor model.^37^

## 5 Conclusion

We demonstrated optical imaging strategies to characterize the metabolic changes of radioresistant and radiosensitive HNSCC cells at a single-cell level under therapeutic stresses. We found that radioresistant and radiosensitive HNSCC cell lines had significantly different metabolic changes in response to radiation stress. The radiation stress also promoted HIF-1α expression in the radioresistant cells. We also found that HIF-1α inhibition during the radiation treatment modulated the metabolic changes induced by radiation stress and radio-sensitizes the radioresistant HNSCC cells, which suggested that the radiation induced HIF-1α overexpression, and the associated metabolic changes may be responsible for the development of the radiation resistance phenotype. Our study demonstrated that optical imaging techniques could be a powerful tool for studying the role of metabolic reprogramming in the development of resistance to cancer therapeutics at the single-cell level in a more efficient, cost-effective, and non-destructive manner.

## Supplementary Material

10.1117/1.BIOS.2.1.012702.s01

## Data Availability

A detailed description of the entire CellProfiler pipeline for microscopy imaging processing is provided in the Supplementary Material. Data and code to generate figures are available: https://github.com/cgzhu123/HNSCC-metabolic-imaging.
