# Supplementary material for: Optical imaging provides flow-cytometry–like single-cell level analysis of HIF-1α-mediated metabolic changes in radioresistant head and neck squamous carcinoma cells
Source: Biophotonics Discov. 2025 Jan 28;2(1):012702. doi: 10.1117/1.BIOS.2.1.012702 (PMC11801402; doi:10.1117/1.BIOS.2.1.012702)
Supplement: Supplementary file 1 [file BIOS_002_012702_SD001.docx]

**Appendix for**

Optical imaging provides flowcytometry-like single cell level analysis of HIF-1α mediated metabolic changes in radio-resistant head and neck squamous carcinoma cells

Jing Yan, ^a,^ † Carlos Frederico Lima Goncalves, ^a,^ † Pranto Soumik Saha, ^a^ Cristina M. Furdui, ^b^ Caigang Zhu ^a, *^

a University of Kentucky, Department of Biomedical Engineering, Lexington, KY 40506, USA

b Wake Forest University, Department of Internal Medicine, Winston-Salem, NC 27157, USA

† These authors have equal contributions to this manuscript.

Correspondence e-mail: [caigang.zhu@uky.edu](mailto:caigang.zhu@uky.edu)

*Imaging Processing Pipeline using Cell Profiler software*

Optical metabolic images were imported into CellProfiler using the **'Images'** module. Metadata extraction was disabled by selecting the option 'No' in the **'Metadata'** module. In the **'NamesAndTypes'** module, the images were processed as 2D images, designated as 'Color Image', and appropriate names were assigned. The intensity range was set according to the image bit-depth, which in this case was 8-bit (0-255).Since automatic object detection requires grayscale images, the **'ColorToGray'** module was used to convert color fluorescence images into grayscale. Using the 'Split' conversion method, the RGB channels of the images were separated. Specifically, the green channel was selected for 2-NBDG (emission peak is ~540 nm), and the red channel was used for TMRE (emission peak is ~590 nm). To expedite the analysis, a random rectangular window measuring 1000 x 750 pixels was cropped from each image using the **'Crop'** module. For individual cell identification from the image, the automated **'IdentifyPrimaryObjects'** module was employed. This tool identifies objects such as cells, cell boundaries, and nuclei within the image. The diameter of the objects was set to range between 50–200-pixel units, discarding objects outside this range as well as those touching the image border. A global minimum cross-entropy thresholding method was applied for identification, with a threshold correlation factor of 1.0. Clumped objects were distinguished based on their intensity profiles. Manual adjustments were then performed using the **'EditObjectsManually'** module, guided by a reference image, to refine and improve the accuracy of cell identification. To analyze the intensity of the identified cells, the **'MeasureObjectIntensity'** module was applied. The **'MeasureObjectIntensityDistribution'** module was used to radially divide the identified cells, setting the number of bins to 4 for detailed distribution analysis.Finally, the analyzed intensity distribution data for individual cells were exported to a spreadsheet using the **'ExportToSpreadsheet'** module for further analysis in MATLAB. By default, CellProfiler outputs intensity values in the range of 0–1. These values were converted to 8-bit format by multiplying them by 255 to match the image bit-depth.


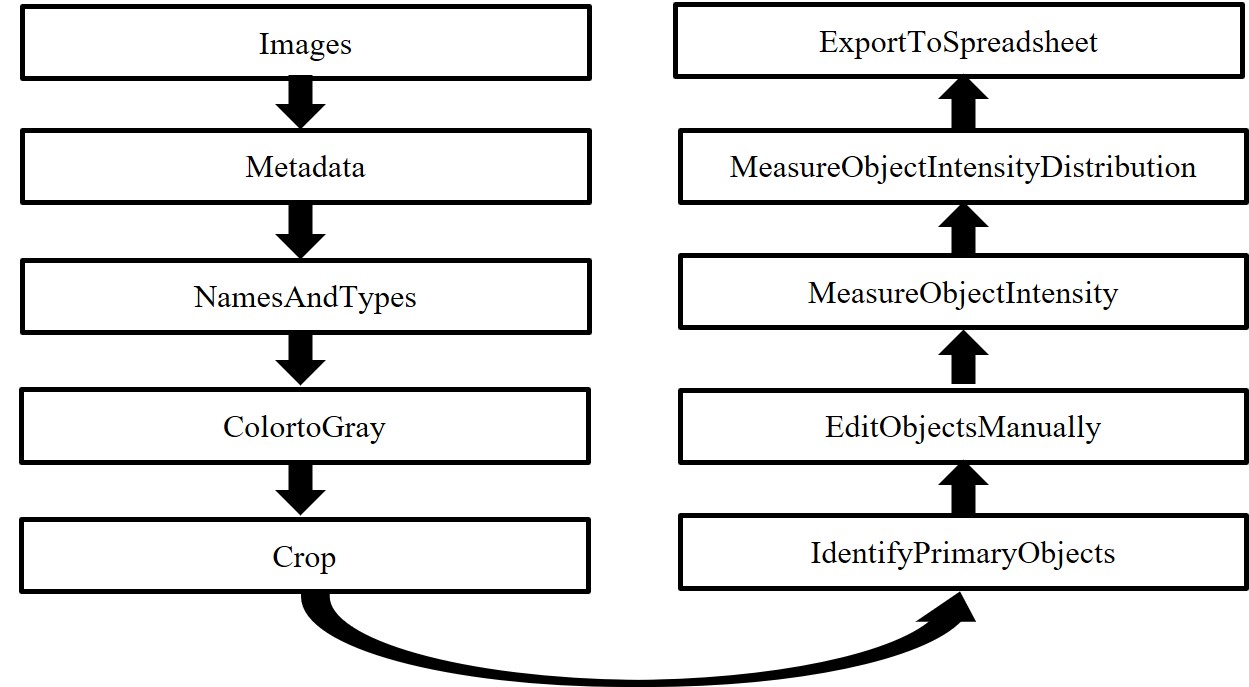


**Figure S1:** Working pipeline of Cell Profiler software for automatic cell identification
